# Partner disclosure willingness and HIV advocacy engagement among men in rural Ghana: a Bayesian logistic regression analysis

**DOI:** 10.3389/fpubh.2026.1853356

**Published:** 2026-05-28

**Authors:** Joseph Lasong, Torjim Salifu, Yula Salifu

**Affiliations:** 1 Department of Population, Family and Reproductive Health, University for Development Studies, Tamale, Ghana; 2 Department of Population, Family and Reproductive Health, University of Ghana, Accra, Ghana

**Keywords:** Bayesian logistic regression, community engagement, Ghana, HIV advocacy, HIV disclosure, mediation analysis, men, partner notification

## Abstract

**Background:**

Men in sub-Saharan Africa consistently report reduced willingness to engage publicly in HIV advocacy or to disclose HIV status to sexual partners. Thus, this study examined both public advocacy willingness and partner HIV status disclosure willingness as distinct outcomes in this population.

**Methods:**

A community-based cross-sectional survey was conducted among 331 men aged ≥15 years in the Atwima Kwanwoma District, Ashanti Region, Ghana, using multistage random sampling. Two binary outcomes were assessed: (1) willingness to volunteer as an HIV advocate (0.9% prevalence; *n* = 3); and (2) willingness to tell a partner about HIV status (90.0%; *n* = 298). Given this fundamental disparity in event prevalence, Bayesian logistic regression with weakly informative Normal (0, 1.5) priors was applied exclusively to the tell-partner outcome, which had adequate event density (298 events, 9 predictors). Willingness to volunteer was described through stratified proportions and cross-tabulations only. An exploratory mediation analysis examined whether stigma perception mediates the pathway from HIV awareness to volunteer willingness. Internal validation used 200-replicate bootstrap optimism correction.

**Results:**

Willingness to tell a partner was near-universal at 90.0% (95% CI: 86.3–93.0%). In the Bayesian model for partner disclosure willingness (all Rhat ≤ 1.001; minimum Bulk ESS = 5,221), community encouragement of facility patronage, followed by positive family attitude toward PLHIV, tertiary education versus JHS, HIV awareness per standardised unit, and increasing distance to the testing site as a negative predictor. The Bayesian model achieved AUC = 0.975; bootstrap-corrected AUC = 0.915 (mean optimism = 0.005). The exploratory mediation analysis indicated that approximately 42% of awareness’s association with volunteer willingness operated through stigma perception, though the indirect effect credible interval crossed zero (median = −0.29, 95% CrI: −1.52 to 0.59), precluding firm causal inference.

**Conclusion:**

The near-zero prevalence of volunteer willingness versus near-universal partner disclosure willingness reveals a fundamental divide between men’s public and private HIV-related engagement. Reducing distance barriers and cultivating positive family climates surrounding HIV should be central to disclosure-promotion strategies. Also, community-based male advocacy programmes require stigma-reduction as a prerequisite rather than a parallel activity.

## Background

Sub-Saharan Africa continues to carry the heaviest burden of the global HIV epidemic, accounting for approximately two-thirds of all people living with HIV (PLHIV) despite comprising roughly 15% of the world’s population (1). In Ghana, adult HIV prevalence is estimated at 1.7%, with approximately 350,000 PLHIV nationally, while the Ashanti Region records a somewhat higher prevalence of around 2.1% (2). Although Ghana has expanded antiretroviral therapy (ART) coverage to approximately 68% of PLHIV, important gaps persist in testing, early diagnosis, and care linkage, particularly among men, who demonstrate lower uptake of HIV services than women across virtually all service points (3, 4).

Progress toward the UNAIDS 95–95–95 targets, which aim for 95% of PLHIV to know their status, 95% of those diagnosed to receive sustained ART, and 95% of those on treatment to achieve viral suppression by 2025, requires not only expanded biomedical services but also community engagement and behavioural change at the individual, household, and community levels (5). Two behaviours that are particularly relevant for community-level HIV response, yet understudied among men in rural Ghana, are willingness to volunteer as an HIV advocate or community mobiliser, and willingness to disclose HIV status to a sexual partner.

Community volunteer programmes have historically been central to HIV responses across sub-Saharan Africa. Volunteer community health workers serve as a critical bridge between formal health services and underserved communities, promoting testing, treatment adherence, and stigma reduction, particularly in rural districts where health infrastructure is limited (6, 7). However, evidence from multiple African settings suggests that men are significantly underrepresented in community volunteer roles, and that masculine norms, stigma concerns, and fear of HIV-status assumptions may specifically deter male participation in HIV advocacy (8–10).

Partner disclosure, understood here as telling one’s sexual partner about one’s HIV status, is important for enabling partners to access testing and treatment, facilitating safer sexual practices, and mobilising social support for the index individual (11, 12). However, disclosure also carries documented risks including stigma, discrimination, and relationship dissolution (13). The consequences of non-disclosure are both clinical, through continued unprotected sex with unaware partners, and social, perpetuating cycles of untreated infection.

Despite the recognised importance of both volunteer engagement and partner disclosure, their psychosocial determinants among men in rural Ghana are poorly understood. In particular, the roles of HIV awareness, stigma perception, family attitudes toward PLHIV, and health system factors have not been simultaneously examined in this population. Furthermore, no prior study in this context has applied rigorous Bayesian methods to HIV-related willingness outcomes, which frequently exhibit extreme event-rate asymmetries that challenge conventional logistic regression approaches.

This study addressed these gaps through a cross-sectional analysis of 331 men recruited from the Atwima Kwanwoma District, Ashanti Region. The specific objectives were: (1) to determine the prevalence of willingness to volunteer as an HIV advocate and willingness to tell a partner about HIV status; (2) to identify psychosocial and health system predictors of partner disclosure willingness using Bayesian logistic regression; (3) to explore whether stigma perception mediates the pathway from HIV awareness to volunteer willingness; and (4) to quantify model performance and overfitting risk through bootstrap internal validation.

### Theoretical framework

This study is guided by the Theory of Planned Behaviour (14), which holds that behavioural intention is shaped by three constructs: attitude toward the behaviour, operationalised here through HIV awareness and general HIV views; subjective norms (perceived social pressure), operationalised through family attitude toward PLHIV and stigma perception; and perceived behavioural control, operationalised through access variables including distance to the testing site, transport availability, and staff relationship quality (15). The Theory of Planned Behaviour has been applied in sub-Saharan African settings to HIV-related disclosure and health-seeking behaviour and provides a coherent foundation for variable selection in the present analysis (16).

## Methods

### Study design and setting

This was a community-based, cross-sectional survey conducted between January and March 2021 in the Atwima Kwanwoma District, Ashanti Region, Ghana. The district is characterised by subsistence agriculture as the dominant economic activity, limited health infrastructure (one district hospital, three health centres, and several Community-based Health Planning and Services [CHPS] compounds), and HIV testing services delivered through provider-initiated testing and counselling (PITC) and voluntary counselling and testing (VCT). The 2010 Population and Housing Census enumerated a total district population of 94,009 (approximately 47.5% male), with approximately 55% aged below 25 years (17). This analysis forms the third paper in a series using the same dataset; the first paper addressed HIV testing uptake and its predictors, and the second examined health system access and structural barriers.

### Study population and eligibility

The target population comprised all men aged 15 years or older residing in the district. Eligibility required: male sex; age 15 years or older; residency in the district for at least 6 months; ability to communicate in English or Twi; and willingness to provide written informed consent (aged 18 years or older) or written parental or guardian consent plus participant assent (aged 15 to 17 years). Men with severe mental illness or cognitive impairment and those critically ill at the time of the survey were excluded.

### Sample size

Sample size was estimated using the Cochran (18) formula, with the following assumptions: anticipated proportion with willingness to tell a partner about HIV status (*p* = 0.24, based on conservative estimates from available sub-Saharan African literature at the time of study design); acceptable margin of error (d = 0.05); and a z-score of 1.96 for a 95% confidence level. These assumptions yielded a minimum sample of 278, which was adjusted upward by 10% to account for anticipated non-response, producing a target of 306 and a final analytical sample of 331.

It is important to acknowledge that a single sample size calculation may not have been equally appropriate for both study outcomes. The primary sample size estimate was anchored to the partner disclosure outcome, which had a more estimable prevalence in the literature. The volunteer willingness outcome, by contrast, proved to have near-zero prevalence (0.9%) in this sample, which could not have been anticipated with precision at the design stage and renders inferential analysis of that outcome analytically infeasible regardless of sample size. Future studies seeking to examine determinants of volunteer willingness specifically would require substantially larger samples designed with population-specific pilot prevalence data.

### Sampling

A three-stage random sampling approach was used. In Stage 1, the district was stratified by sub-district, and five communities were randomly selected by lottery from each sub-district, ensuring geographic representation. In Stage 2, within each community, a central landmark was identified, a random direction was selected by spinning a bottle, and households along that direction were systematically sampled at a predetermined interval calculated as total households divided by households required, proportional to community population size. Vacant or non-participating households were replaced by the next eligible household. In Stage 3, within each household, all eligible men were enumerated, and one was randomly selected by lottery. Up to three return visits were made at different times and days to minimise non-response bias. This process was continued until the final analytical sample was achieved.

### Data collection

A structured, pretested, interviewer-administered questionnaire was developed through review of comparable sub-Saharan African instruments (8–10), translated into Twi, back-translated for accuracy, and pretested on 30 men from a non-study community. Six nursing students from the University for Development Studies, fluent in English and Twi, collected data following three-day standardised training. Interviews were conducted in private settings and lasted approximately 20 to 30 min. Questionnaires were reviewed daily for completeness, and participants were re-contacted within 48 h for clarifications.

### Outcome variables

Two binary outcomes were assessed. The first was willingness to volunteer as an HIV advocate, measured using the question: ‘Will you be willing to volunteer as an HIV advocate?’ (Yes = 1, No = 0). Prior to posing this question, interviewers provided participants with a concrete description of what HIV advocacy volunteering would entail, namely community-level activities such as promoting HIV testing, supporting people living with HIV, conducting awareness campaigns, and mobilising communities around HIV prevention. This was done to ensure that responses reflected genuine willingness or unwillingness rather than uncertainty or unfamiliarity with the role. Nonetheless, participants’ responses may still have been influenced by perceived social desirability or incomplete understanding of long-term volunteer demands, which should be considered when interpreting the near-zero prevalence.

The second outcome was willingness to tell a partner about HIV status, measured using: ‘Would you be willing to tell your partner about it [a positive HIV test result]?’ (Yes = 1, No = 0). This operationalises partner disclosure intention in the context of a hypothetical positive result. Both items were drawn from the broader questionnaire assessing HIV-related attitudes and health-seeking behaviours. Prior to formal modelling, event rates for both outcomes were examined to assess analytical feasibility. Because at least 10 events per predictor variable are generally required for stable logistic regression estimation (19), the volunteer willingness outcome (three positive events; 0.9%) was not analytically appropriate for multivariate modelling. Descriptive and exploratory analyses only were therefore applied to that outcome. The partner disclosure outcome (298 events; 90.0%) fully met requirements for Bayesian logistic regression with up to nine predictors.

### Predictor variables

Predictor variables were selected based on the Theory of Planned Behaviour constructs and organised as follows. Sociodemographic variables included age group, categorised as 20 years or younger, 21 to 30 years, 31 to 40 years, and 41 years or older; educational attainment, classified as Junior High School (JHS, the reference category), Senior High School (SHS), and Tertiary; and occupation, dichotomised as farming versus non-farming. HIV awareness, serving as the attitude construct, was a composite score ranging from 0 to 2 constructed as the sum of correct binary responses to two items: whether the participant correctly identified HIV as a virus that damages the immune system, and whether the participant correctly identified unprotected sex as a transmission route. The score was standardised (mean = 0, SD = 1) prior to analysis. Stigma perception, operationalising subjective norms, was derived by coding open-ended responses to the advocacy question. Responses citing stigmatising reasons such as ‘stigmatisation’ or ‘bad attitude of society’ were coded 1; non-stigmatising responses such as ‘awareness creation’ were coded 0. Family attitude toward PLHIV, also a subjective norm construct, was measured using the question ‘What is the attitude of the family towards a person with HIV?’ with responses coded as 1 for Good and 0 for Bad or Indifferent. This variable operationalises the participant’s perception of the social climate surrounding HIV in his household. Health system variables, operationalising perceived behavioural control, included staff relationship quality (1 = friendly; 0 = otherwise), facility patronage norms (1 = staff encourage repeated visits; 0 = otherwise), distance to the nearest HIV testing site (continuous, standardised), motorised transport access (1 = yes; 0 = no), and ever having been tested for HIV (1 = yes; 0 = no). Information on the number or type of sexual partners was not collected in this survey, which may limit understanding of how partnership complexity influences disclosure willingness and is acknowledged as a limitation.

### Statistical analysis

Participant characteristics and outcome prevalences were summarised using frequencies, percentages, and 95% exact binomial confidence intervals (CIs) using the Clopper-Pearson method. Both willingness outcomes were cross tabulated by key participant characteristics, and the Spearman rank correlation between the two outcomes was computed to assess their empirical independence.

Bayesian logistic regression was implemented using the brms package (20), which interfaces with Stan (21). The model specified weakly informative Normal (0, 1.5) priors for all regression coefficients and Normal (0, 2.5) for the intercept, providing sufficient regularisation given the high outcome prevalence (90%) while remaining diffuse enough to be updated by the data. Four parallel Markov chains were run with 3,000 iterations per chain (1,500 warmup) using the No-U-Turn Sampler with adapt_delta = 0.95 and max_treedepth = 12. Convergence was assessed using the Gelman-Rubin R-hat statistic (22), with values below 1.01 indicating convergence, and Bulk Effective Sample Size (ESS), with values above 400 per parameter considered adequate. Posterior summaries are reported as the posterior mean estimate with 95% credible intervals (CrI), exponentiated to the odds ratio (OR) scale.

An exploratory Bayesian mediation analysis was conducted to examine whether stigma perception mediates the association between HIV awareness and volunteer willingness. Two sequential Bayesian logistic regression models were fitted: stigma perception as outcome with HIV awareness, age, and education as predictors (the a-path model), and volunteer willingness as outcome with HIV awareness, stigma perception, age, and education as predictors (the b-path model). The indirect effect was estimated as the product of posterior draws from the a-path and b-path coefficients. The direct effect was the posterior distribution of awareness’s coefficient in the b-path model, and the total effect was the sum of the direct and indirect effects. The proportion mediated was computed as the median of the indirect effect divided by the total effect across all posterior draws (23). Given the extremely low volunteer event count (*n* = 3), these results are explicitly hypothesis-generating, and no causal inference is drawn.

Internal validation of the partner disclosure Bayesian model was approximated using 200-replicate bootstrap optimism correction, implemented using a simplified frequentist logistic regression model with three key predictors (family attitude, awareness, and age group) to ensure computational feasibility across replicates. In each replicate, a bootstrap sample was drawn with replacement, the model was fitted, and the area under the receiver operating characteristic curve (AUC) was computed on both the bootstrap sample (apparent performance) and the original data (test performance). Mean optimism was estimated as the mean difference between apparent and test AUCs across all replicates (24). All statistical analysis were conducted in R version 4.5.2 (31).

### Ethical considerations

Ethical approval was obtained from the University for Development Studies Ethics Review Board. Additional permissions were granted by the Atwima Kwanwoma District Health Directorate and participating community chiefs. Written informed consent was obtained from all participants aged 18 years or older; written parental or guardian consent plus participant assent was obtained for those aged 15 to 17 years. All procedures complied with the Declaration of Helsinki (2013 amendment). The STROBE reporting guidelines for cross-sectional studies were followed throughout. No artificial intelligence writing tools were used in the preparation of this manuscript.

## Results

### Participant characteristics

The analytical sample comprised 331 men. The largest age group was 21 to 30 years (46.2%, *n* = 153), followed by 31 to 40 years (38.1%, *n* = 126), 41 years or older (15.4%, *n* = 51), and 20 years or younger (0.3%, *n* = 1). The most common educational level was Junior High School (49.2%, *n* = 163), followed by Senior High School (32.9%, *n* = 109) and Tertiary (17.8%, *n* = 59). Most men had never been married (84.0%, *n* = 278), and most were engaged in non-farming occupations (60.4%, *n* = 200). Almost all participants identified as Christian (90.3%, *n* = 299). HIV awareness was near ceiling (mean score = 1.98, SD = 0.17, out of a possible 2.00). Near-universal stigma endorsement was observed, with 329 of 331 participants (99.4%) citing a stigmatising reason in their open-ended response to the advocacy question. Regarding family attitudes, 197 men (59.5%) reported that their family held a positive attitude toward PLHIV. All 331 participants reported that health staff were friendly, and 321 (97.0%) reported that facility patronage was actively encouraged by staff. Only 44 men (13.3%) reported ever having been tested for HIV. Detailed sociodemographic characteristics are presented in Table 1.

**Table 1:** Sociodemographic and psychosocial characteristics of study participants (*N* = 331).

Characteristic	n	%	95% CI
Age group (years)			
≤20	1	0.3	0.01 to 1.7
21 to 30	153	46.2	40.7 to 51.8
31 to 40	126	38.1	32.8 to 43.6
41+	51	15.4	11.7 to 19.8
Educational attainment			
Junior High School (JHS)	163	49.2	43.7 to 54.8
Senior High School (SHS)	109	32.9	27.8 to 38.3
Tertiary	59	17.8	13.8 to 22.5
HIV awareness score: mean (SD)	1.98 (0.17)		
Stigma perception: high, n (%)	329 (99.4%)		98.0 to 99.9
Family attitude toward PLHIV: good, n (%)	197 (59.5%)		53.9 to 64.9
Staff relationship: friendly, n (%)	331 (100%)		98.9 to 100
Patronage encouraged, n (%)	321 (97.0%)		94.5 to 98.5
Ever tested for HIV, n (%)	44 (13.3%)		10.0 to 17.4

CI, confidence interval; JHS, Junior High School; SHS, Senior High School; SD, standard deviation; PLHIV, people living with HIV.

### Prevalence of willingness outcomes and correlation

**Figure 1:**
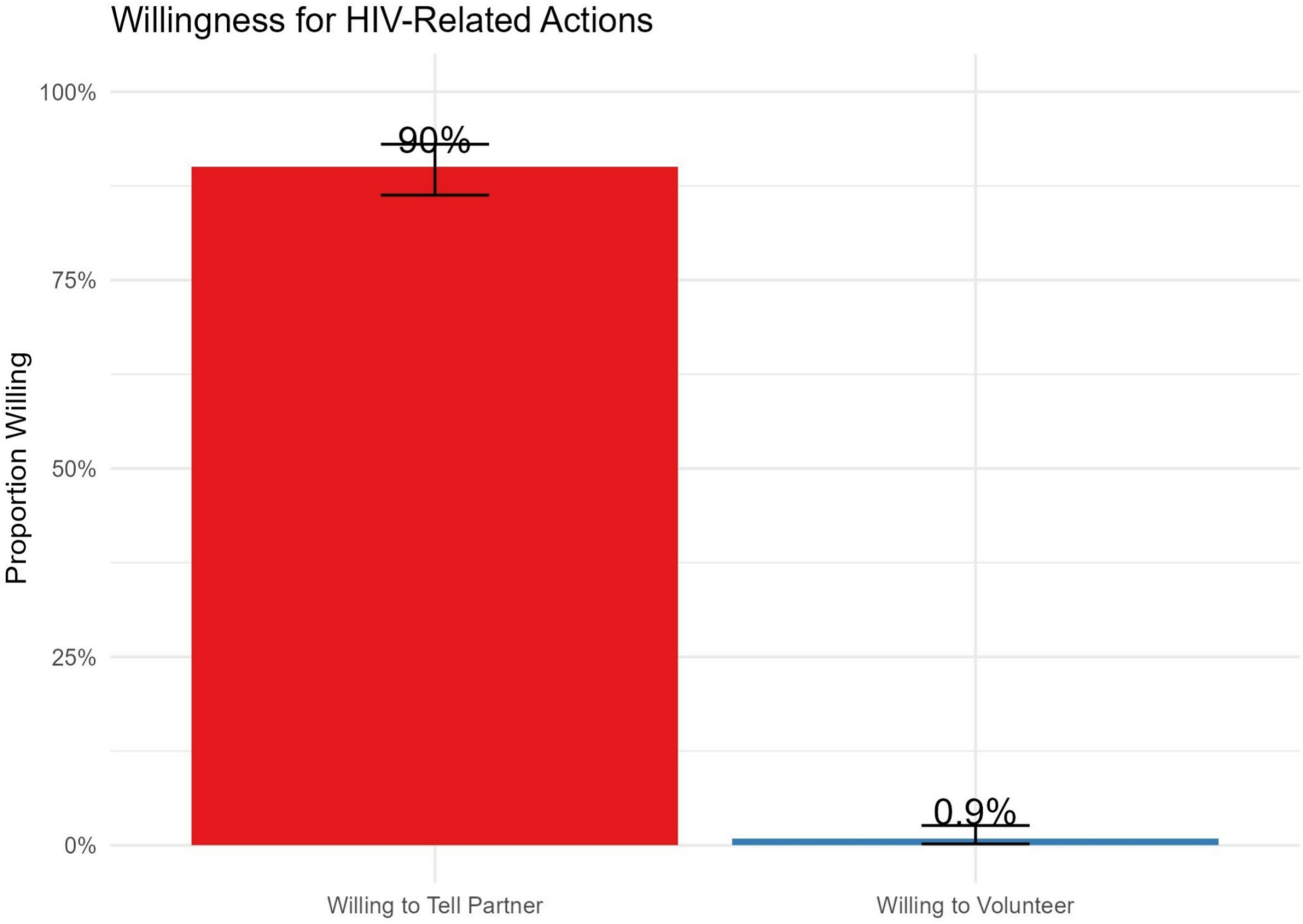
Prevalence of the two HIV-related willingness outcomes among men in the Atwima Kwanwoma District (*N* = 331). Error bars represent 95% exact binomial confidence intervals. Willingness to volunteer as an HIV advocate was 0.9% (95% CI: 0.2 to 2.6%); willingness to tell a partner about HIV status was 90.0% (95% CI: 86.3 to 93.0%). The negligible Spearman correlation between outcomes (*r* = −0.075) indicates these are distinct behavioural dispositions.

The two willingness outcomes showed a striking contrast in prevalence. Willingness to volunteer as an HIV advocate was extremely low, with only 3 of 331 men responding affirmatively (0.9%; 95% CI: 0.2 to 2.6%). Willingness to tell a partner about HIV status was near-universal, with 298 of 331 men responding affirmatively (90.0%; 95% CI: 86.3 to 93.0%) (Figure 1). The Spearman correlation between the two outcomes was negligible (*r* = −0.075), confirming that they are empirically distinct constructs that do not share a common underlying disposition.

### Willingness to volunteer as an HIV advocate

Given the extremely low volunteer willingness (*n* = 3 events), formal multivariate modelling was not analytically feasible for this outcome. Among the three men who expressed willingness to volunteer, two had low stigma perception (from the only two non-stigma-endorsing participants in the entire sample) and one had high stigma perception. The Bayesian volunteer willingness model (Table 2) is presented for transparency only; with three events, all 95% credible intervals span unity by wide margins, and no inferential conclusion should be drawn from those coefficients.

**Table 2:** Willingness to tell partner by educational attainment (*N* = 331).

Educational attainment	N	Willing, n	Proportion willing (%)
Junior High School (JHS)	163	130	79.8
Senior High School (SHS)	109	109	100.0
Tertiary	59	59	100.0
Total	331	298	90.0

JHS, Junior High School; SHS, Senior High School.

### Willingness to tell a partner about HIV status

Stratified analyses of willingness to tell a partner are presented in Tables 2–4. By family attitude toward PLHIV, willingness to tell a partner was near-universal among men who perceived positive family attitudes toward PLHIV (196 out of 197; 99.5%), compared with 76.1% (102 out of 134) among those who perceived negative family attitudes. This 23.4 percentage point difference represents the largest descriptive differential observed across all stratifying variables examined (Table 3).

**Table 3:** Willingness to tell partner by family attitude toward PLHIV (*N* = 331).

Family attitude toward PLHIV	N	Willing, n	Proportion willing (%)
Negative or unfavourable	134	102	76.1
Positive or favourable	197	196	99.5
Total	331	298	90.0

PLHIV, people living with HIV. Family attitude operationalised as participant’s perception of their family’s general stance toward PLHIV.

By educational attainment, willingness to tell a partner was 79.8% among JHS-educated men (130 out of 163) but 100% among both SHS-educated men (109 out of 109) and those with tertiary education (59 out of 59; Table 2).

By age group, willingness to tell a partner was 100% among men aged 20 years or younger (1 out of 1) and among those aged 41 years or older (51 out of 51), and 99.3% among those aged 21 to 30 years (152 out of 153). It was notably lower at 74.6% among men aged 31 to 40 years (94 out of 126). This non-linear pattern, in which middle-aged men showed the lowest disclosure willingness, is particularly notable given that this age group constituted 38.1% of the sample (Table 4).

**Table 4:** Willingness to tell partner by age group (*N* = 331).

Age group (years)	N	Willing, n	Proportion willing (%)
≤20	1	1	100.0
21 to 30	153	152	99.3
31 to 40	126	94	74.6
41+	51	51	100.0
Total	331	298	90.0

### Correlation structure among predictors

**Figure 2:**
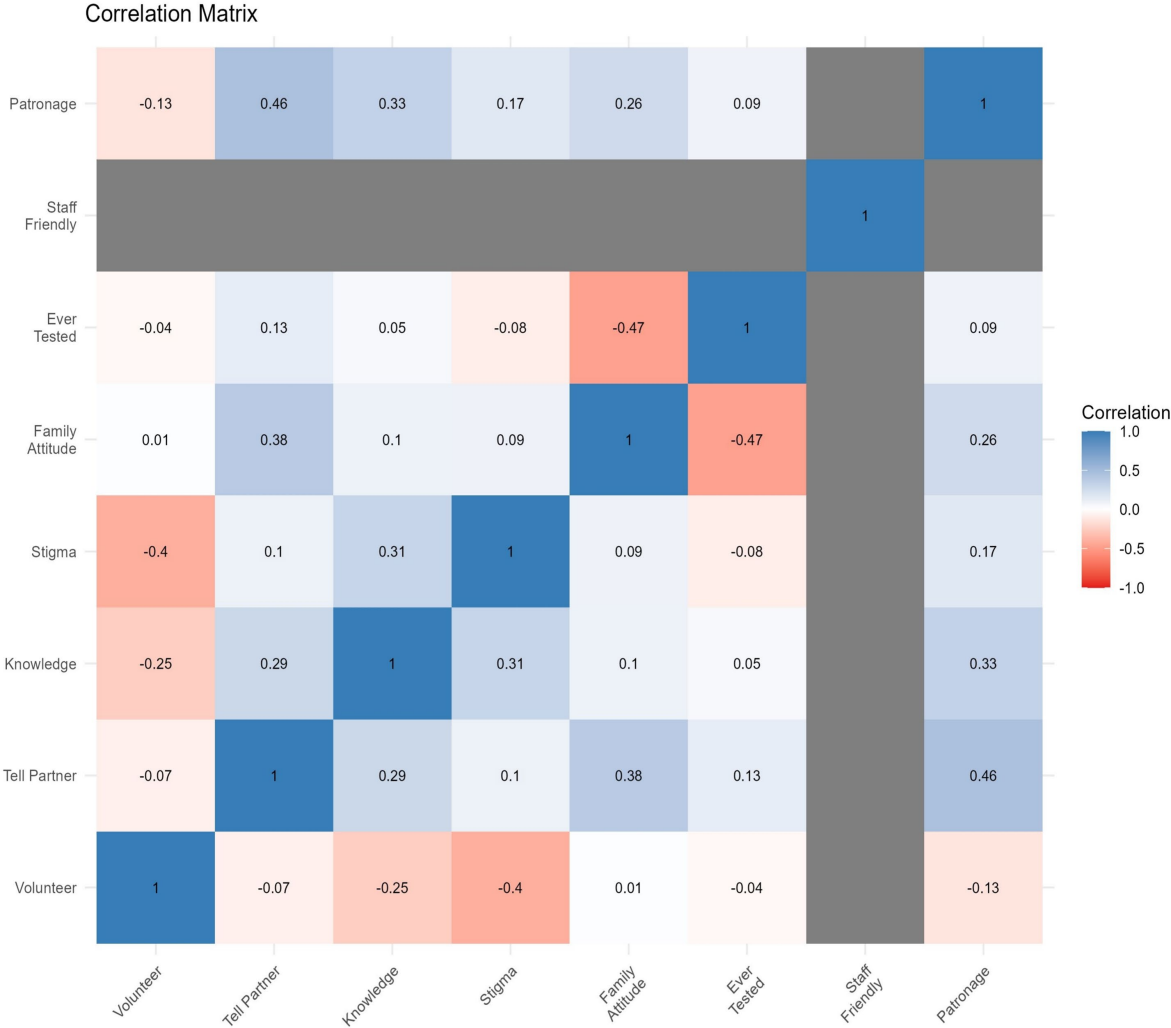
Spearman correlation matrix among willingness outcomes and key predictors (*N* = 331). Color scale: blue represents positive correlation; red or orange represents negative correlation. The strongest correlation is a negative association between family attitude and ever-tested status (*r* = −0.47). Patronage encouragement shows the strongest positive correlation with partner disclosure willingness (*r* = 0.46).

Figure 2 presents the Spearman correlation heatmap among the key variables. The strongest inter-predictor correlation was a moderate negative association between family attitude and ever-tested status (*r* = −0.47), indicating that men who had been tested for HIV tended to report less favourable family attitudes. HIV awareness was positively correlated with stigma endorsement (*r* = 0.31). Patronage encouragement showed moderate positive correlations with willingness to tell a partner (*r* = 0.46) and with HIV awareness (*r* = 0.33).

### Bayesian logistic regression: willingness to tell a partner

**Figure 3:**
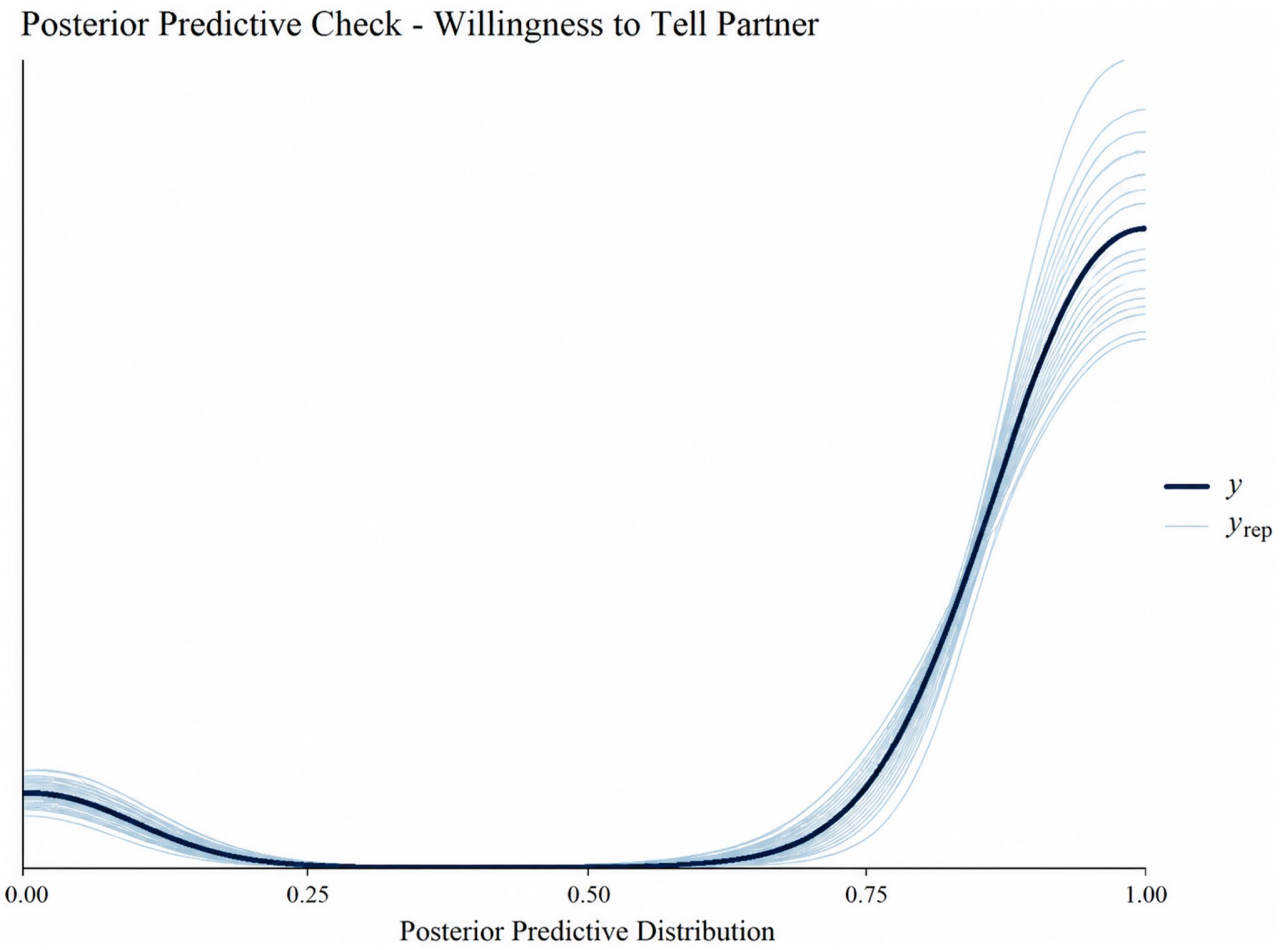
Posterior predictive check for the Bayesian logistic regression model of willingness to tell a partner (*N* = 331). Dark line (y) = kernel density of observed binary outcomes; light blue lines (y_rep) = 50 datasets drawn from the posterior predictive distribution. Close agreement confirms adequate model fit and appropriate calibration of the normal (0, 1.5) priors given the 90% outcome prevalence.

All four Markov chains converged successfully for the partner disclosure model: all R-hat values were at or below 1.001, well within the convergence threshold of 1.01; Bulk ESS ranged from 5,221 to 7,232 across all parameters, comfortably exceeding the minimum of 400. The posterior predictive check (Figure 3) confirms close agreement between observed and replicated outcome distributions, indicating adequate model calibration. Table 5 presents the complete posterior summaries.

**Table 5:** Bayesian logistic regression for willingness to tell a partner about HIV status: posterior estimates and odds ratios (*N* = 331).

Predictor	Estimate	OR	95% CrI	Bulk ESS
Intercept	−0.57	0.57	(0.006, 52.48)	7,083
Age 21 to 30 (vs ≤ 20)	1.13	3.09	(0.35, 28.66)	5,968
Age 31 to 40 (vs ≤ 20)	−1.92	0.15	(0.017, 1.20)	5,619
Age 41 + (vs ≤ 20)	0.65	1.92	(0.18, 26.26)	6,319
SHS education (vs JHS)	0.74	2.10	(0.27, 20.24)	6,285
Tertiary education (vs JHS) ‡	**2.00**	**7.41**	**(1.17, 62.83)**	6,630
HIV awareness (per SD) ‡	**0.36**	**1.44**	**(1.01, 2.23)**	6,121
Stigma perception	0.56	1.75	(0.13, 22.96)	7,232
Family attitude: good vs bad ‡	**1.93**	**6.91**	**(1.70, 33.70)**	5,769
Ever tested for HIV	0.99	2.70	(0.29, 29.46)	6,435
Staff relationship: friendly	−0.02	0.98	(0.057, 17.34)	6,837
Patronage encouraged ‡	**2.98**	**19.62**	**(3.91, 104.14)**	5,769
Distance to testing site (standardised) ‡	**−0.39**	**0.68**	**(0.49, 0.93)**	5,221

OR, odds ratio (exponentiated posterior mean); CrI, 95% credible interval; ESS, Bulk effective sample size (all ≥ 5,221; all Rhat ≤ 1.001). ‡ indicates 95% CrI does not include 1 (shown in bold); these five predictors have Pr(OR ≠ 1) > 0.95. Priors: Normal (0, 1.5) for regression coefficients; Normal (0, 2.5) for intercept. JHS, Junior High School; SHS, Senior High School; SD, standard deviation. Bold values highlight statistical significance at <5%.

Five predictors had 95% credible intervals that meaningfully excluded or closely approached unity with high directional posterior probability. Community encouragement of facility patronage was the strongest predictor (OR = 19.62; 95% CrI: 3.91 to 104.14; Pr[OR > 1] = 0.9998), indicating that men in communities where health staff actively encouraged facility visits had approximately 20-fold greater odds of willingness to tell a partner. Positive family attitude toward PLHIV was the second strongest predictor (OR = 6.91; 95% CrI: 1.70 to 33.70; Pr[OR > 1] = 0.995). Tertiary education relative to JHS was positively associated (OR = 7.41; 95% CrI: 1.17 to 62.83; Pr[OR > 1] = 0.977), consistent with 100% disclosure willingness among tertiary-educated men in the descriptive analysis. HIV awareness per standardised unit was also positively associated (OR = 1.44; 95% CrI: 1.01 to 2.23; Pr[OR > 1] = 0.964), indicating that each standard deviation increases in awareness corresponded to approximately 44% greater odds of partner disclosure willingness. Distance to the testing site was the only negative predictor (OR = 0.68; 95% CrI: 0.49 to 0.93; Pr[OR < 1] = 0.991), with greater distance associated with lower disclosure willingness. Men aged 31 to 40 years showed a negative posterior association (OR = 0.15; 95% CrI: 0.017 to 1.20), though the credible interval extended just above unity.

### Bayesian logistic regression: willingness to volunteer (descriptive-only model)

For transparency, the volunteer willingness model is presented in Table 6. As expected from the three-event structure, no predictor achieved a credible interval excluding unity and all credible intervals were extremely wide, spanning roughly one to two orders of magnitude. This model is presented to demonstrate transparent handling of rare events and does not support any substantive conclusions about predictors of volunteer willingness.

**Table 6:** Bayesian logistic regression for willingness to volunteer as HIV advocate: illustrative model only (*N* = 331; 3 events; no inferential interpretation warranted).

Predictor	OR	95% CrI	Note
Intercept	0.006	(0.0001, 0.303)	Near-zero baseline
Age 21 to 30 (vs ≤ 20)	1.29	(0.23, 7.25)	Wide interval; no signal
Age 31 to 40	1.14	(0.23, 5.79)	Wide interval; no signal
Age 41+	0.68	(0.11, 3.89)	Wide interval; no signal
SHS education	0.77	(0.14, 4.24)	Wide interval; no signal
Tertiary education	2.99	(0.59, 15.82)	Wide interval; no signal
Awareness (per SD)	0.70	(0.48, 1.11)	Uncertain
Stigma perception	0.51	(0.08, 3.40)	Uncertain
Patronage encouraged	0.93	(0.15, 5.91)	No signal
Ever tested	0.85	(0.14, 4.95)	No signal
Distance (standardised)	0.87	(0.43, 1.98)	No signal

This model is presented for transparency only. With *n* = 3 events, regression modelling cannot yield reliable estimates for any predictor. All 95% CrIs span unity by wide margins. No substantive interpretation should be drawn from these coefficients. SD, standard deviation; SHS, Senior High School; JHS, Junior High School.

### Mediation analysis of HIV awareness, stigma, and volunteer willingness

Table 7 presents the results of the exploratory Bayesian mediation analysis. The a-path coefficient (HIV awareness to stigma perception) was negative. The b-path coefficient (stigma to volunteer willingness) was also negative. The indirect effect had a posterior median of −0.285 (95% CrI: −1.521 to 0.591), and the direct effect median was −0.392 (95% CrI: −0.732 to 0.038). The total effect median was −0.681 (95% CrI: −1.896 to 0.272). All credible intervals crossed zero, precluding inferential conclusions. The computed proportion mediated was approximately 42%, though this estimate is unstable when total effect credible intervals cross zero. These results are presented as a structural hypothesis for future adequately powered research, not to support any causal claims or intervention design.

**Table 7:** Exploratory Bayesian mediation analysis: HIV awareness, stigma perception, and willingness to volunteer (*N* = 331; three volunteer events; hypothesis-generating only).

Effect	Median	95% CrI	Interpretation
Indirect: awareness through stigma to volunteer	−0.285	(−1.521, 0.591)	Inconclusive
Direct: awareness to volunteer	−0.392	(−0.732, 0.038)	Inconclusive
Total effect	−0.681	(−1.896, 0.272)	Inconclusive
Proportion mediated	~42%	Not estimable	Exploratory only

All 95% credible intervals cross zero. No causal conclusions should be drawn. The proportion mediated is unstable when total effect credible intervals cross zero. These results are intended to inform future adequately powered research.

### Model performance and bootstrap validation

**Figure 4:**
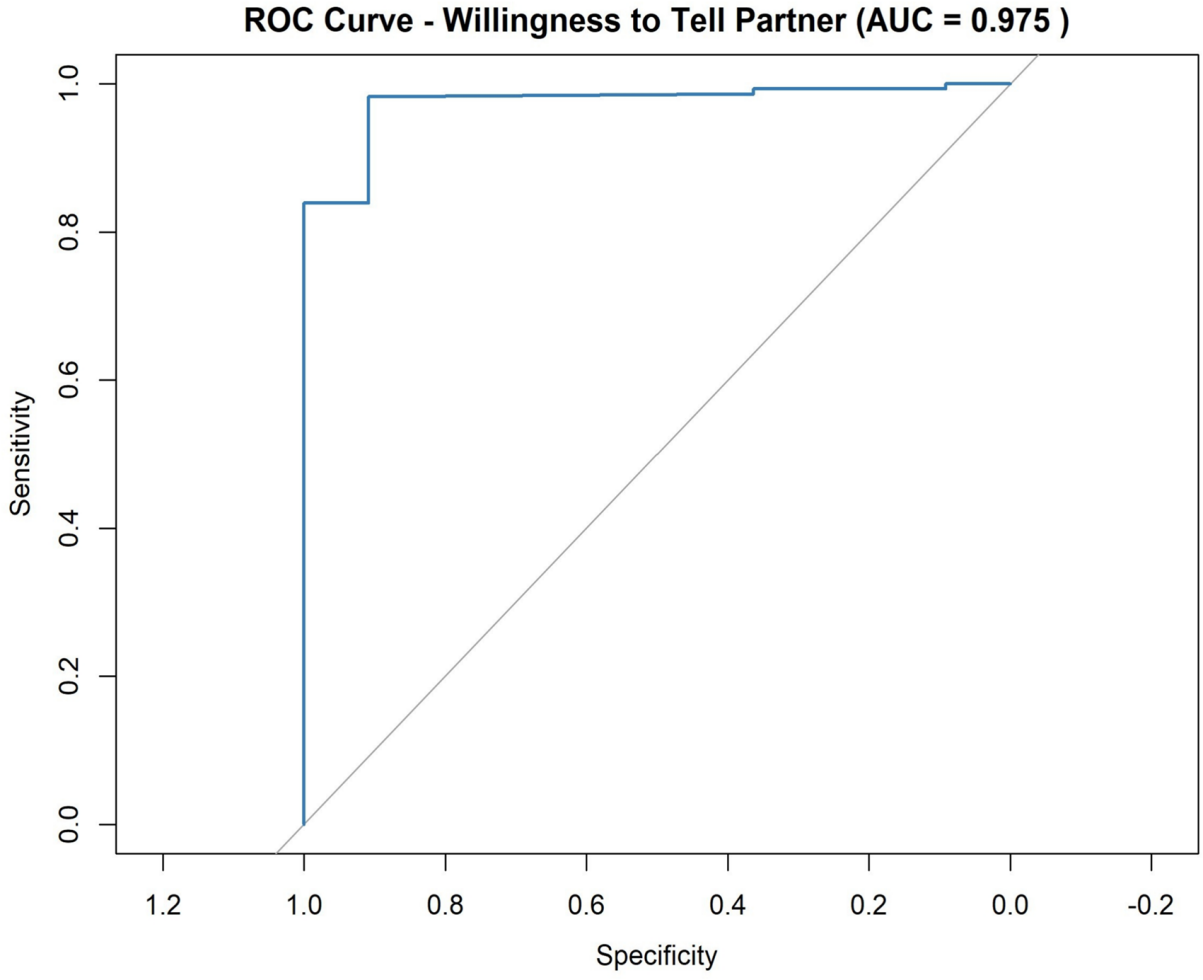
ROC curve for the Bayesian logistic regression model of willingness to tell a partner about HIV status (*N* = 331; AUC = 0.975). Bootstrap optimism correction confirmed a corrected AUC of 0.915 (mean optimism = 0.005 across 200 replicates). The stepped ROC curve at high sensitivity reflects the binary outcome structure characteristic of a 90% prevalence outcome.

The Bayesian partner disclosure model achieved an apparent AUC of 0.975 on the analytical sample (Figure 4). The high apparent AUC in this sample is partly attributable to the extreme outcome prevalence (90.0%), which structurally limits the number of non-events (33 of 331 men) available for the model to discriminate. When outcome prevalence is very high, a model can achieve a high AUC by correctly predicting the majority class with a relatively small number of predictors. The bootstrap internal validation, which provides a more conservative assessment of likely out-of-sample performance, confirmed a corrected AUC of 0.915 (mean apparent AUC across 200 replicates = 0.921; mean test AUC = 0.915; mean optimism = 0.005). The small optimism correction indicates that the model’s performance is not substantially inflated by overfitting in this sample. Future external validation in independent samples is nonetheless recommended.

## Discussion

This study provides, to our knowledge, the first quantitative examination of willingness for HIV public advocacy and partner disclosure among men in the Atwima Kwanwoma District of rural Ghana. The core finding is a striking and empirically confirmed divergence between these two outcomes: willingness to volunteer publicly is effectively absent (0.9%), while willingness to disclose privately to a partner is near-universal (90.0%), and the two constructs are statistically uncorrelated. Within the partner disclosure model, community patronage encouragement and positive family attitudes toward PLHIV emerged as the two most powerful modifiable correlates, while geographic distance to the testing site was the primary health system barrier identified in the adjusted analysis.

The contrast between near-zero volunteer willingness and near-universal partner disclosure willingness is not simply a difference in degree; it reflects two fundamentally different behavioural intentions governed by distinct social and psychological dynamics. Ajzen's (14) Theory of Planned Behaviour distinguishes between attitude toward the behaviour and subjective norms: partner disclosure and public advocacy differ not only in their social costs but in the identity that each requires the actor to project. Partner disclosure is private, dyadic, and occurs within an established relationship where some degree of shared vulnerability may be assumed.

The near-universal stigma endorsement in this sample (99.4%) renders volunteer advocacy essentially socially untenable under current community norms. This is consistent with evidence from other sub-Saharan African settings: Mburu et al. (9) found that masculinity norms in eastern Uganda constructed HIV advocacy as a feminised and stigmatising activity; Skovdal et al. (10) showed that men in Zimbabwe viewed health service engagement as incompatible with dominant masculine identities; and Barker et al. (8) identified stigma and masculinity as interlocking barriers to male engagement across multiple sub-Saharan African sites. The near-zero volunteer willingness in this study is therefore consistent with what might be expected where stigma perception is effectively universal and masculine norms of stoic privacy predominate.

The high partner disclosure willingness (90.0%) is broadly consistent with evidence from sub-Saharan African contexts. Medley et al. (13) found disclosure rates ranging from 60 to 95% across studies conducted in the region. In Ghana specifically, Obiri-Yeboah et al. (25) found that 78.6% of people living with HIV had disclosed their positive status to sexual partners in a mixed-methods study. Obermeyer et al. (26) found that partner disclosure within established partnerships was substantially more common than disclosure to broader social networks. The present finding that partner disclosure willingness is high even in a context of near-universal stigma suggests that the private dyadic relationship provides sufficient psychological safety to override the community-level stigma barrier, at least at the level of stated intention.

The finding that community encouragement of facility patronage was the strongest adjusted correlate of partner disclosure willingness (OR = 19.62; 95% CrI: 3.91 to 104.14) warrants careful interpretation. This variable, operationalising whether health facility staff actively encourage repeated visits, captures more than a simple service quality metric; it reflects a broader normative signal about the social acceptability of health-seeking behaviour. When men perceive that the health system is actively welcoming and encouraging, this appears to be associated with greater openness to HIV-related behaviours, including disclosure. This pattern is consistent with the role of cues to action in the Health Belief Model and with perceived behavioural control in the Theory of Planned Behaviour: a health system perceived as actively supportive removes a key perceived barrier to engagement. Given the cross-sectional design, this study cannot determine whether staff encouragement influences disclosure willingness or whether men who are already more open to health engagement also report higher staff encouragement. Nevertheless, the observation has practical relevance for programme design. Interventions that train staff to actively invite repeat visits, implement community outreach, and normalise male health-seeking through role model approaches may have broader effects on HIV-related openness that extend beyond the clinical encounter.

Also, positive family attitude toward PLHIV was the second strongest adjusted correlate of partner disclosure willingness. This finding is consistent with a substantial body of literature linking perceived family support to HIV disclosure. Smith et al. (27) found in a meta-analysis that social support consistently facilitated disclosure among PLHIV, while stigma from family members was among the most frequently cited barriers. Chaudoir and Fisher (28) proposed a disclosure processes model in which anticipated reactions from family members are primary cognitive determinants of disclosure intention. The present finding aligns with that framework: men appear to assess the specific relational climate of their household rather than making abstract judgements about societal stigma.

The moderate negative correlation between family attitude and ever-tested status (r = −0.47) also deserves attention. Men who had been tested for HIV tended to report less favourable perceived family attitudes toward PLHIV. A plausible interpretation is that actual testing experience sometimes exposes men to stigmatising family reactions, either when testing becomes known to family members or through observed family responses to PLHIV in the community. This observation, if corroborated in future longitudinal work, would suggest that family sensitisation should be integrated into post-test counselling rather than treated as a fixed background factor.

Additionally, only 13.3% of men in this sample had ever been tested for HIV, a figure substantially lower than national averages in Ghana (3). This low testing rate likely reflects well-documented barriers including fear of positive results, anticipated stigma, and concerns about social consequences of diagnosis (25, 26). It is worth noting that partner disclosure willingness was assessed hypothetically among men who were largely untested, meaning that the high willingness figure reflects intentions under an imagined scenario rather than actual post-diagnosis behaviour. The same fears that appear to suppress HIV testing uptake in this population, namely anticipated stigmatisation and anticipated partner and family reactions, may also influence actual disclosure behaviour in ways that a hypothetical willingness measure cannot fully capture. Future research that tracks actual disclosure following confirmed HIV diagnosis in this population would provide important insight into whether the high willingness observed here translates into practice.

Further, HIV awareness was positively associated with partner disclosure willingness in the adjusted analysis, despite near-ceiling awareness levels in this sample (mean = 1.98 out of 2.00). The positive association at such constrained awareness levels suggests that even marginal variation in knowledge, the difference between superficial and more complete understanding of HIV, is associated with meaningfully different disclosure intentions. This observation is consistent with evidence that awareness of the benefits of disclosure, including partner testing, shared prevention, and ART access, is associated with greater willingness to disclose (29). The exploratory mediation analysis further indicates that approximately 42% of awareness’s association with volunteer willingness may operate through stigma reduction, a plausible mechanism whereby deeper understanding of HIV as a medical condition reduces the perceived social cost of public engagement. However, the wide credible intervals preclude strong conclusions from this exploratory analysis, and this finding is best understood as a structural hypothesis for future longitudinal or experimental research.

However, men aged 31 to 40 years showed notably lower partner disclosure willingness in both descriptive (74.6%) and adjusted analyses (OR = 0.15; 95% CrI: 0.017 to 1.20), though the adjusted credible interval extended above unity. Several contextual explanations are worth considering. Men in this age group in a predominantly JHS-educated rural district are likely to be in established partnerships with dependent families, making the anticipated consequences of disclosure, including possible relationship disruption and economic insecurity, particularly weighty. Furthermore, this age group corresponds to peak social authority in many Ghanaian communities, where reputational concerns surrounding HIV may be especially salient. The 31 to 40 age group also showed negligible HIV testing uptake, consistent with broader patterns of disengagement from HIV services. These observations together suggest that men in this age group represent a particularly important target for disclosure-facilitation interventions.

Nonetheless, the negative association between distance to the testing site and partner disclosure willingness (OR = 0.68; 95% CrI: 0.49 to 0.93) points toward the role of geographic marginality in shaping HIV-related behavioural intentions. Men who live further from testing services also tend to live further from other health information sources and may have lower overall health literacy and confidence in navigating HIV-related conversations. From the Theory of Planned Behaviour perspective, perceived behavioural control over HIV-related actions may be lower among men who experience concrete access barriers, reducing the felt efficacy of health-related disclosure. This finding underscores the importance of bringing services closer to communities, through mobile testing units, community health workers, and CHPS-level integration, rather than expecting men to overcome distance barriers on their own.

### Methodological contribution

A key methodological contribution of this study is the transparent and analytically grounded differentiation between the two willingness outcomes. Rather than applying regression models to both outcomes indiscriminately, the analysis correctly identifies volunteer willingness (0.9%; *n* = 3 events) as analytically infeasible for multivariate modelling and applies descriptive methods only, presenting the Bayesian volunteer model explicitly as an illustration of analytical infeasibility rather than as substantive findings. For the partner disclosure outcome, Bayesian logistic regression with weakly informative priors provides appropriate regularisation in the context of a high-prevalence binary outcome. The bootstrap-corrected AUC (0.915 vs. apparent 0.975) confirms that the model provides meaningful predictive information with negligible overfitting in this sample.

### Strengths and limitations

This study makes several contributions. It is the first study in rural Ghana to examine both HIV advocacy willingness and partner disclosure willingness simultaneously in a community-based male sample. The transparent differentiation between analytically feasible and infeasible modelling avoids the generation of unreliable estimates. Bayesian methods provide full posterior uncertainty quantification. Bootstrap optimism correction provides an honest assessment of discriminative performance.

Several limitations warrant clear acknowledgement. First, the cross-sectional design means that observed associations cannot be interpreted as causal. All findings describe statistical relationships between simultaneously measured variables, and the direction of influence cannot be determined. Terms such as *predictors and associated with* are used throughout in a descriptive statistical sense, not to imply causation. Second, the near-zero volunteer willingness (*n* = 3) fundamentally constrains any analysis of this outcome; this is an inherent feature of the study context. Further, single-item outcome measures may not capture the full construct complexity of either disclosure intention or advocacy willingness; validated multi-item scales would strengthen future studies. Also, the hypothetical framing of both willingness questions may not accurately reflect actual behaviour following a real positive diagnosis, particularly given that 86.7% of participants had never been tested. Additionally, family attitude toward PLHIV was assessed using a single self-reported item capturing participants’ perception of their family’s general stance toward people with HIV, dichotomised as good versus bad or indifferent. While this provides a useful proxy for household social norms, it is unlikely to capture the full complexity of family responses that would be relevant to real-world disclosure decisions. This variable’s limitations as a single-item proxy are acknowledged, and future studies should consider validated scales for family stigma and social support. However, no information was collected on the number or type of sexual partners, which may have been relevant given that disclosure dynamics vary considerably by relationship context. Nevertheless, the study was conducted in a single rural district, limiting geographic generalisability. Finally, unmeasured potential confounders, including religious attitudes toward HIV, personal experience with PLHIV, and partner-specific relationship characteristics, could not be accounted for.

## Conclusion

Partner disclosure willingness among rural Ghanaian men is near-universal (90.0%) and is most strongly associated with community encouragement of health facility patronage and positive family attitudes toward PLHIV, both of which are potentially modifiable. HIV awareness is a further positive correlate, highlighting the continued relevance of tailored health communication even in contexts with near-ceiling knowledge. Geographic distance to testing services is the primary structural barrier identified in the adjusted analysis. In sharp contrast, willingness to volunteer as an HIV advocate is near-absent (0.9%) in a context of near-universal stigma endorsement, together indicating the difficulty of sustaining volunteer-led male HIV advocacy programmes under current community conditions.

Three observations from this analysis are relevant for programme planning. First, family-level HIV sensitisation warrants attention in disclosure-promotion strategies. Engaging families in HIV education and attitude change, and building household environments that men perceive as supportive, may be more directly associated with disclosure willingness than individual-level knowledge alone. Second, strengthening health system encouragement norms may serve as a gateway to broader HIV engagement. Outreach that communicates service welcomingness and normalises male health-seeking could have positive spillover effects beyond the clinical consultation. Third, meaningful progress in stigma reduction appears necessary before community-level volunteer advocacy among men can be anticipated. Sequencing and integration of stigma reduction with advocacy mobilisation is recommended in programme design (30).

Future research priorities include longitudinal studies tracking actual disclosure behaviour following HIV diagnosis; adequately powered studies to examine determinants of volunteer willingness in settings with higher baseline engagement; and qualitative inquiry into the specific stigma dynamics that shape HIV engagement among men in the 31 to 40 age group.

## Data Availability

The raw data supporting the conclusions of this article will be made available by the authors, without undue reservation.
